# Molecular Characterization of *Leptospira* spp. in Environmental Samples from North-Eastern Malaysia Revealed a Pathogenic Strain, *Leptospira alstonii*

**DOI:** 10.1155/2016/2060241

**Published:** 2016-04-03

**Authors:** Muhammad Azharuddin Azali, Chan Yean Yean, Azian Harun, Nurul Najian Aminuddin Baki, Nabilah Ismail

**Affiliations:** ^1^School of Agriculture Science and Biotechnology, Faculty of Bioresources and Food Industry, Universiti Sultan Zainal Abidin, Tembila Campus, 22200 Besut, Terengganu, Malaysia; ^2^Department of Medical Microbiology and Parasitology, School of Medical Sciences, Health Campus, Universiti Sains Malaysia, 16150 Kubang Kerian, Kelantan, Malaysia

## Abstract

The presence of pathogenic* Leptospira* spp. in the environment poses threats to human health. The aim of this study was to detect and characterize* Leptospira* spp. from environmental samples. A total of 144 samples comprised of 72 soil and 72 water samples were collected from markets and recreational areas in a north-eastern state in Malaysia. Samples were cultured on Ellinghausen and McCullough modified by Johnson and Harris media. Leptospires were positive in 22.9% (*n* = 33) of the isolates. Based on partial sequences of 16S rRNA, a pathogenic leptospire,* Leptospira alstonii* (*n* = 1/33), was identified in 3% of the isolates followed by intermediate leptospire (*L. wolffii*, *n* = 1/33, and* L. licerasiae*, *n* = 7/33) and nonpathogenic leptospire,* L. meyeri* (*n* = 22/33) in 24.2% and 66.7%, respectively. This study demonstrates the presence of a clinically significant pathogenic* L. alstonii* in the environments which could pose health risks to the occupants and visitors.

## 1. Introduction

Leptospirosis is one of the most important zoonoses infecting both developing and developed countries in the world [1]. Traditionally, leptospirosis was classified as an occupational disease [2]. Later, leptospirosis was also associated with recreational activities through exposure to the contaminated soil or water [3–5]. An increasing number of people involved in outdoor activities have increased the chances of infection. Pets and rodents are the sources of infection in the housing areas. High density of rats in the markets poses potential threats to visitors and town service workers [6]. The expansion of housing areas also increases the opportunistic contact between humans and the infected wildlife. Environments are frequently associated with nonpathogenic leptospires. However, a novel pathogenic species,* L. kmetyi*, has been isolated from environmental samples in Malaysia [7].

Malaysia is a tropical country with high seasonal rainfall, warm temperatures, and wet and humid climate. These conditions lengthen the survival of leptospires in the environment. It is common that floods occur following heavy rainfall during monsoon season in the East Coast. The presence of leptospires in the environment during flooding may potentially cause an outbreak of leptospirosis. Kelantan which is located in the East Coast of Malaysia is among the affected states. The number of cases increased during flooding from on average 20 cases to 31 cases [8]. Many inhabitants in this state are also involved in agricultural activities which pose the highest risk as 50 cases were reported from the workers in this sector [8]. It was also reported that, in 2011, one death was recorded out of 276 cases [9]. Previously, one death was reported from a waterfall in Kelantan which is Jeram Pasu [10]. In order to identify the potential threats of leptospirosis in the environments, the present study aims to detect and characterize* Leptospira* spp. isolated from the selected environments in Malaysia.

## 2. Material and Methods

### 2.1. Sampling Sites

**Figure 1:**
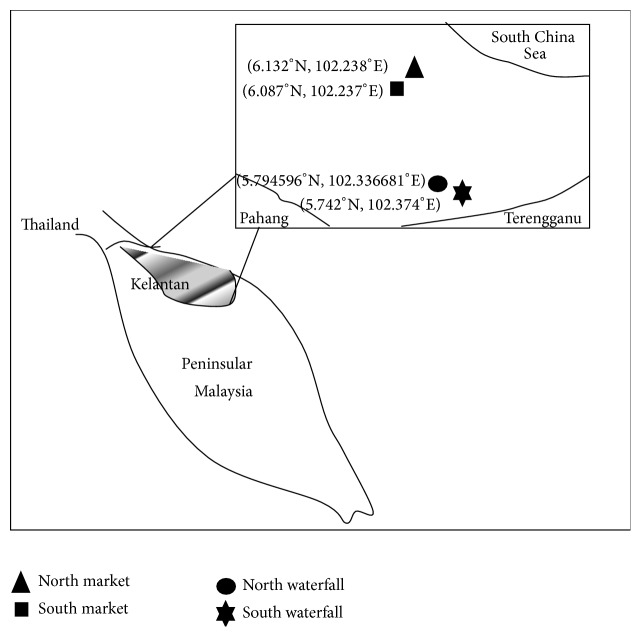
Location of sampling sites in north-eastern state of Malaysia.

Samplings were conducted from December 2012 to November 2013 at the selected recreational areas (waterfalls with spots of running and still water) and markets in the north-eastern state of Malaysia, Kelantan (Figure 1). Those sampling sites, which cover urban and rural areas of three districts, were selected based on previous reports of leptospirosis cases, rat infestations, and improper waste management.

### 2.2. Isolation of Leptospires

#### 2.2.1. Sample Collection

Water samples in recreational areas were collected from shaded, suspected areas for the presence of animals and in-between rock areas. Water samples were collected about 1 foot below the water surface. Water samples were filtered through 0.2 *μ*m Nalgene® Filter Unit and 40 mL of the samples were transferred into centrifuge tubes and then centrifuged at 4000 ×g, 27°C for 20 mins. Two mL of the samples were inoculated into 5 mL of liquid EMJH media with the addition of 5-fluorouracil (100 *μ*g/mL). For soil samples, sampling locations were selected from wet and shaded areas, from garbage sites, and in the places where spoiled food was spotted. In sterile 50 mL falcon tubes, the soil samples were mixed with sterile water and shaken vigorously. The suspension was allowed to settle for 5–10 minutes before being filtered using 0.2 *μ*m Nalgene® Filter Unit.

#### 2.2.2. Culture of Leptospires

Several drops of the filtrates were inoculated into EMJH media supplemented with antimicrobial agents (5-fluorouracil, 100 *μ*g/mL) and incubated at 30°C in a shaker incubator at 25 rpm to accelerate the growth of leptospires. The presence of leptospires was examined under dark-field microscopy using 20x and 40x magnification daily for 28 days. Leptospires can be distinguished by other spirochetes based on their characteristic thin helical structures with prominent hooked ends and motility.

#### 2.2.3. Culture Purification

In an event of contamination, 1000 *μ*L of contaminated cultures were transferred into fresh liquid EMJH media supplemented with sulfamethoxazole and trimethoprim (40/8 *μ*g/mL), amphotericin B (5 *μ*g/mL), and 5-fluorouracil (100 *μ*g/mL) according to Chakraborty et al., with some modifications [14]. The concentration of trimethoprim was changed from 20 *μ*g/mL to 8 *μ*g/mL and fosfomycin was not added to the media. The cultures were examined under dark-field microscopy using 20x and 40x magnification daily for 28 days. If the contaminants were still present, the cultures were diluted in sterile distilled water using a serial dilution technique starting from dilutions 10^−3^, 10^−6^, 10^−9^, and 10^−12^ and then transferred into solid EMJH media. The diluted culture was incubated for 3 weeks or until the leptospires colonies were observed on plates. Single, isolated colonies were transferred from the plates into liquid media using sterile Pasteur pipettes. The cultures were examined under dark-field microscopy daily for 28 days.

### 2.3. Serological Characterization

The microscopic agglutination test (MAT) was performed using a set of hyperimmune rabbit antisera purchased from the Queensland Health Clinical and Statewide Services, Australia, including Autumnalis, Tarassovi, Pyrogenes, Javanica, Grippotyphosa, Copenhageni, Canicola, Ballum, Hardjoparjitno, Celledoni, Patoc, Djasiman, Icterohaemorrhagiae, Pomona, Australis, and Hebdomadis using the available method [15]. Cultures of 4 to 7 days of age were used as antigens. The turbidity of the cultures was adjusted to 0.5 McFarland. The plates were gently shaken to mix contents, covered to exclude debris and prevent evaporation, and incubated at 30°C for 2 hours. All of the isolates were screened for agglutination at titre ≥1 : 100 under dark-field microscope. Agglutination of at least 50% of the leptospires was considered as positive.

### 2.4. Molecular Characterization of the Isolates

DNA extraction was performed on isolates by using Qiagen DNeasy® Blood & Tissue Kit (Qiagen, USA) according to the manufacturer's protocols for Gram negative bacteria and stored at 20°C until use. A 20 *μ*L-PCR reaction mixture containing 1x PCR buffer, 2.5 mM MgCl_2_, 0.16 mM dNTP's premixed, 0.04 *μ*M of each primer, and 0.75 units of Taq Polymerase was used in all PCR amplifications. Amplification was performed in MJ Research thermocycler with an initial denaturation at 94°C for 5 minutes, followed by 30 cycles of 94°C for 30 seconds, annealing for 30 seconds at Ta of the primers (Table 1), and extension at 72°C for 30 seconds. The cycles were then followed by the final extension at 72°C for 5 minutes. Primers G1/G2 and B64-I/B64-II were used to detect the* Leptospira* spp. Primer targeting virulence gene was used for the detection of* lipL32* gene. Bak2 primer pair, targeting 16SrRNA gene, was used for species identification. The primer sets Sapro 1 and Sapro 2 were used to detect the nonpathogenic leptospires. Amplified products were characterized by electrophoresis of 5 *μ*L of each reaction on 1.5% agarose gel for 50 min at 90 V. The PCR product amplified by Bak2 primer pair was purified using QIAquick PCR Purification Kit (Qiagen, USA) and subsequently sequenced using Sanger sequencing kits and instruments supplied by Applied Biosystems®, US, performed by First Base Laboratories (Selangor, Malaysia). The DNA sequences were edited in BioEdit [16] and compared against the GenBank database using BLAST. The 16S rRNA gene partial sequences of all isolates were aligned with the 16S rRNA sequences of the type strains obtained from GenBank by using Multiple Sequence Comparison by Log-Expectation (MUSCLE) in MEGA 6 software [17]. The annotated sequences were submitted to the GenBank.

## 3. Results

### 3.1. Isolation of Leptospires

A total of 144 samples comprised of 72 water (markets, *n* = 36; recreational areas, *n* = 36) and 72 soil (markets, *n* = 36; recreational areas, *n* = 36) samples were collected. Out of those samples, 33 were positive for leptospires based on their characteristic morphology and motility. Among positive samples, 7 (5%) were water samples and 26 (18%) were soil samples. A total of 29 positive samples (water, *n* = 7; soil, *n* = 22) were collected from markets and only four positive samples (water, *n* = 0; soil, *n* = 4) were collected from recreational areas.

### 3.2. Serological Characterization

All of the leptospiral isolates in this study were not agglutinated with any of the reference hyperimmune sera used in MAT (Table 2). Hence, the isolates did not belong to any of the tested serogroups.

### 3.3. Molecular Characterization

Out of 33 isolates, 18 isolates were detected as* Leptospira* spp. using G1/G2 and B64-I/B64-II primers (Table 2). None of the isolates were found to contain the virulence gene. Molecular identification by 16S rRNA verified that, from 33 positive samples in this study, 31 isolates were identified as* Leptospira* spp. and two isolates were identified as leptospiral closest relatives,* Leptonema illini*. A pathogenic leptospire,* L. alstonii* (3%), was isolated from a soil sample in a market. Eight isolates (24.2%) were identified as intermediate pathogenic species comprised of seven* L. wolffii* and one* L. licerasiae*. A total of 22 isolates (66.7%) were identified as nonpathogenic leptospires,* L. meyeri*.

**Figure 2:**
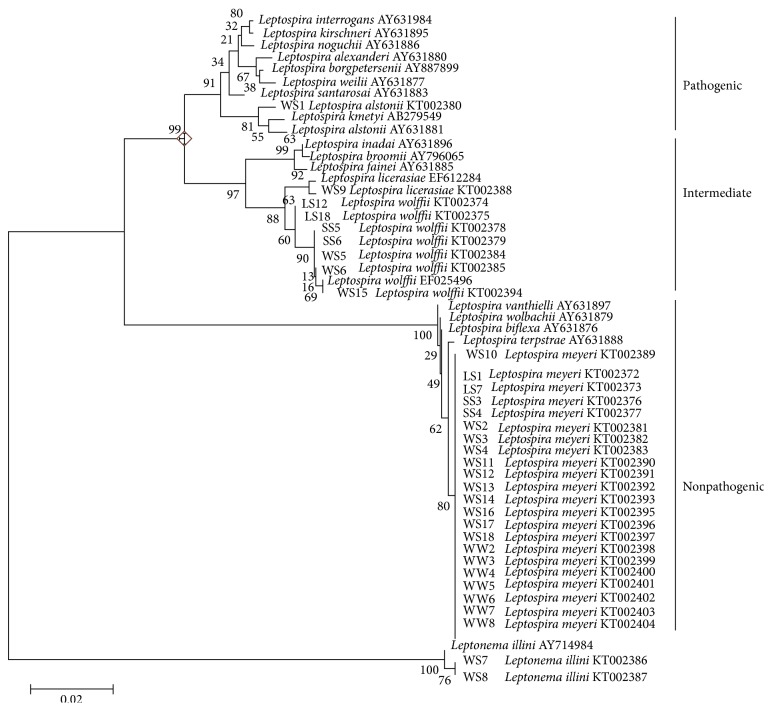
A phylogenetic tree generated by the Neighbour-Joining method using MEGA 6 based on 16S rRNA gene sequence of* Leptospira* isolates and the selected strains.

The phylogenetic tree constructed using the Neighbour-Joining method was illustrated in Figure 2.* Leptospira* spp. isolated in this study were clearly separated into three clades. Strain WS1 was discovered from soil samples included in the pathogenic clade. It was closely related to type strains of* L. alstonii* and* L. kmetyi*. LS12, LS18, SS5, SS6, WS5, WS6, WS9, and WS15 were grouped into intermediate pathogenic clade. LS1, LS7, SS4, WS1, WS2, WS3, WS4, WS10, WS11, WS12, WS13, WS14, WS16, WS17, WS18, WW2, WW3, WW4, WW5, WW6, WW7, and WW8 were grouped into nonpathogenic clade. These isolates were closely related to type strains of* L. meyeri* and* L. yanagawa*.

## 4. Discussion

Even though this is not the first study reporting the detection and characterization of leptospiral isolates from environmental samples, its isolation from recreational and market areas would give an impact to the community. Ganoza et al. reported that the prevalence of leptospires in water samples from markets was 67.9% [18]. In the present study, positive water samples from markets were found in 19.4% only. Leptospires may be unable to survive in the drainage water in this market. The water was found to contain detergent and appeared oily, most probably due to its close proximity to the nearby food stalls. It has been reported that 30 ppm of detergent (Ceepryn, Fixanol, and Sapamine) were lethal to leptospires in water in 5 minutes [19]. The prevalence of leptospires in water samples from recreational areas in Malaysia was 11.67% [20]. In the current study, a lower prevalence of 5.56% was noted, which could be due to the effect of inappropriate choice of sampling points. The chosen points may have not been exposed to the leptospiral contamination.

All isolates recovered in this study showed negative reactions to 16 reference hyperimmune sera tested in MAT. Similarly, negative results of MAT for environmental isolates were also reported from another study in which eight isolates from the selected urban sites in Malaysia were negative for a total of 25 hyperimmune sera tested [20]. The availability of the reference hyperimmune sera was very limited because none of local reference laboratories supplies the hyperimmune sera. Since the sera used in this study were procured overseas, the available panel of sera may not cover the circulating local serovar. A total of 37 serovars of* Leptospira* from 13 serogroups have been identified in Malaysia [21]. This highlights the importance of having locally produced hyperimmune sera available for local use.

In our study, G1/G2 and B64-I/B64-II primers detected 18 out of 33 isolates as* Leptospira* spp. However, 16S rRNA gene sequencing identified 31 out of 33 isolates as* Leptospira* spp. G1/G2 and B64-I/B64-II primers were not able to detect all strains of* Leptospira* spp. The occurrence of new strains of leptospires in humans, animals, and environment has increased the need for new primers that are more specific than G1/G2 and B64-I/B64-II. Leptospiral putative virulence gene,* LipL32*, rises as a new target for the detection of pathogenic leptospires though its role in virulence mechanisms remains unknown [22]. This is further complicated by the absence of this gene in pathogenic and intermediate leptospires isolated in this study. This gene was either absence or undetectable by PCR because in some cases the* LipL32* gene was not detectable in* L. licerasiae* by PCR but its product was detectable by both Southern blot hybridization and Western immunoblot [23]. In the previous study by Murgia et al., primer sets Sapro 1 and Sapro 2 were used to detect the saprophytic leptospires [11]. However, these primers were found to be unspecific [11] as pathogenic leptospire,* L. alstonii*, was amplified by this primer in this study.

In this study, a pathogenic species,* L. alstonii*, was isolated from a soil sample in market area. Our finding was in line with previous studies that also isolated* L. alstonii* from the environment [24]. Exposure to the soil in market areas which harboured pathogenic strains may explain the high seropositivity of leptospiral antibodies among garbage collectors and town cleaners in Kelantan [6].* L. alstonii* was possibly harboured by frogs as it was initially isolated from frogs [25]. The isolation of leptospires from frogs specifically from kidney was reported as early as 1964 [26]. Several pathogenic serovars or serogroups such as bim, Australis, and Ballum have been isolated from frogs [27, 28]. However, inoculation of frogs with pathogenic serovars to establish experimental leptospirosis led to unsatisfactory results as leptospires were not recovered in their organs [29].

The intermediate species,* L. wolffii* and* L. licerasiae*, were isolated from markets and recreational areas. They are able to cause diseases in humans although less frequent [12]. The presence of pathogenic and intermediate species warrants adherence to precautions and preventive measures among the visitors to those areas. It was reported that Malaysians who were involved in recreational activities especially water related are 2.4 times more likely to acquire leptospirosis compared to those who were not involved in similar activities [30]. Leptospirosis was detected in wildlife in Malaysia including wild mammals-monkeys, bats, squirrels, and mongoose [31]. Those animals could be the reservoirs for leptospires in recreational areas located in remote areas.

A previous study reported high numbers of* L. meyeri* (88.1%) from environmental samples [24]. Our study also demonstrated the predominance of* L. meyeri* in Malaysia environment. Though this species was nonpathogenic, there is controversy surrounding* L. meyeri* pathogenicity status.* L. meyeri* serovar ranarum ICF which was previously considered as nonpathogenic was shown to be related to pathogenic strain suggesting that this species consisted of both pathogenic and nonpathogenic strains [32]. This strain was also amplified by sets of primers specific for pathogenic* Leptospira* and not the sets of primers specific for saprophytic strain [11]. This result is also supported by phylogenetic analysis in another study [13]. This disagreement demands a novel typing method of* Leptospira* spp. which covers not only pathogenic species but also intermediate and nonpathogenic strains to resolve the uncertainties in species' designation in the genus of* Leptospira*. The accurate species designation and classification are crucial because the presence of clinically imperative leptospires in the environment would not be overlooked.

Phylogenetic analysis of the studied isolates using 16S rRNA gene sequences concurred with the findings of previous report by Morey et al. in 2006 that the pathogenic, intermediate, and nonpathogenic species each formed one clade [33]. Intermediate groups were closely related to the pathogenic rather than nonpathogenic group. Isolates in the nonpathogenic group were closely related to both* L. meyeri* and* L. yanagawa*. It was reported that species within the nonpathogenic groups were separated by no more than 10 bp [33]. Using the 16S rRNA gene,* L. meyeri* recovered in this study were not separated. In order to demonstrate intraspecies genetic variations, the use of other gene loci is necessary, either singly or in combination. It was also suggested that a cut-off point of 1,000 base pairs (bp) was applied for leptospiral species identification [34]. The phylogenetic analysis of environmental isolates using 16S rRNA was also found to be better than using PFGE profiles.

## 5. Conclusion

The data presented in this study demonstrates the presence of a clinically significant pathogenic* L. alstonii* and the predominance of* L. meyeri* in the environment. The pathogenic strain was found to not contain one of the highly conserve putative leptospiral virulence genes. Early prevention is required to reduce the risks of infection amongst the population. Hence, continuous control and surveillance are essential in lowering the burden of the disease.

## Figures and Tables

**Table 1:** List of primers used.

Primer	Targeted genes	Annealing temperature (°C)	Product size	Sequences	Sources
G1	*secY*		285 bp	5′-CTGAATCGCTGTATAAAAGT 3′	[11]
G2		55		5′-GGAAAACAAATGGTCGGAAG 3′	
B64-I	*flaB*		563 bp	5′-CTGAATTCTCATCTCAACTC 3′	
B64-II				5′-GCAAAATCAGATGGACG AT 3′	

Pathogenic gene	*LipL32*	58	120 bp	5′-AGG TCT TTACAGAATTTCTTTCA 3′	This study
				5′-TTACTTAGTCGCGTCAGAA 3′	

Sapro 1	*rrs*	55	240 bp	5′-AGAAATTTGTGCTAATACCGAATGT 3′	[12]
Sapro 2				5′-GGCGTCGCTGCTTCAGGCTTTCG 3′	

Bak2-F	16S rRNA	52	796 bp	5′-AGTTTGATCMTGGCTCAG 3′	[13]
Bak2-R				5′-GGACTACHAGGGTATCTAAT 3′	

**Table 2:** Results for molecular and serological tests.

Species (*n* = 33)	G1/G2; B64-I/B64-II	SAPRO1/SAPRO2	Virulence gene (*LipL32*)	MAT
	(*n*)	(*n*)		
*L. alstonii *(*n* = 1)	1	1	—	—
*L. wolffii *(*n* = 7)	—	7	—	—
*L. licerasiae *(*n* = 1)	—	1	—	—
*L. meyeri *(*n* = 22)	17	22	—	—
*Leptonema illini *(*n* = 2)	—	2	—	—

Total (33)	18	33	0	0
